# Identification of genes associated with human-canine communication in canine evolution

**DOI:** 10.1038/s41598-022-11130-x

**Published:** 2022-06-09

**Authors:** Akiko Tonoike, Ken-ichi Otaki, Go Terauchi, Misato Ogawa, Maki Katayama, Hikari Sakata, Fumina Miyasako, Kazutaka Mogi, Takefumi Kikusui, Miho Nagasawa

**Affiliations:** 1 Department of Veterinary Science, Azabu University, 1-17-71, Fuchinobe, Chuo-ku, Sagamihara, Kanagawa 252-5201 Japan; 2 Center for Human and Animal Symbiosis Science, Azabu University, 1-17-71, Fuchinobe, Chuo-ku, Sagamihara, Kanagawa 252-5201 Japan

**Keywords:** Evolution, Evolutionary genetics

## Abstract

The dog (*Canis familiaris*) was the first domesticated animal and hundreds of breeds exist today. During domestication, dogs experienced strong selection for temperament, behaviour, and cognitive ability. However, the genetic basis of these abilities is not well-understood. We focused on ancient dog breeds to investigate breed-related differences in social cognitive abilities. In a problem-solving task, ancient breeds showed a lower tendency to look back at humans than other European breeds. In a two-way object choice task, they showed no differences in correct response rate or ability to read human communicative gestures. We examined gene polymorphisms in oxytocin, oxytocin receptor, melanocortin 2 receptor, and a Williams–Beuren syndrome-related gene (WBSCR17), as candidate genes of dog domestication. The single-nucleotide polymorphisms on melanocortin 2 receptor were related to both tasks, while other polymorphisms were associated with the unsolvable task. This indicates that glucocorticoid functions are involved in the cognitive skills acquired during dog domestication.

## Introduction

Domestic dogs (*Canis lupus familiaris*) are the oldest and one of the most popular species of companion animals^1,2^. Research has uncovered that their closest relatives are wolves^3,4^, and that modern dog breeds were domesticated into human society over 10,000 years ago^5^. However, their origins and the process of domestication have yet to be fully understood.

Domestication involves the selection of traits that fundamentally alter wild species to become more useful to humans^6^. Dogs have been closely associated with humans for many centuries and there are now more than 400 breeds of domestic dogs^7^. Dogs are thought to have been selected for human proximity^8^, a wide range of physical traits^9^, behaviour, and more broadly, the ability to build social relationships^10^.

Research comparing the behaviour of dogs and wolves has indicated that dogs show less avoidance and aggression toward familiar humans^11^. In addition, dogs show better understanding of human communicative signals than wolves^12,13^ and, in some studies, dogs have even demonstrated an ability to form social bonds with humans^10^. Although the leading force behind dog domestication remains unclear, it is certain that these behavioural adaptations, including docility and the ability to form social bonds with humans, are important factors which enabled dogs to be incorporated into human society.

Animal behaviour, particularly social behaviour, is modulated and influenced by the actions of various hormones in the brain. For example, glucocorticoid is widely known as a hormone which is positively related to anxiety and social avoidance^14,15^. The role of glucocorticoid in the domestication process has been proposed in many studies. One example is the domestication experiment on silver foxes (*Vulpes vulpes*), in which domesticated foxes were selectively bred for non-aggressiveness or fear toward humans. They showed reduced cortisol secretion and exhibited greater exploratory behaviour in, and tolerance of, new environments^16^. Another hormone that is of interest is oxytocin (OT). OT is known to play an important role in the bonding and attachment between mother and young in mammalian species, as well as in other types of relationships, such as in pair bonding^17,18^. Recently, the study of OT-mediated bonding has been expanded to interspecies relationships, including those between dogs and humans^10^. OT was also shown to have a function in enabling dogs to respond to human social cues, such as pointing^19^. Moreover, the marked variations in OT receptor (OTR) genes between purebred dogs of different breeds, free-ranging dog populations, wolf subspecies, and golden jackals have suggested that the OTR gene could have been a target gene during domestication^20^.

Dog breeds show differences in temperament and variations in emotionality, vocalization, activity, problem solving, reactions to human handling, trainability^21,22^, and the ability to understand human communicative signals^23^. As wolves, which share the same ancestors, do not show good human-communication skills, genetic changes should have occurred during the domestication of dogs, and the behavioural differences between dogs and wolves should appear as different phenotypes, depending on the genetic backgrounds of different dog breeds^24^.

Owing to remarkable advances in technology, it has become possible to study the genetic relationships between domestic dog breeds to construct a genetic classification for them^25,26^. There appear to have been two population bottlenecks during the dog domestication process, one likely associated with their initial domestication from wolves to dogs and the other with the recent formation of modern dog breeds^3,27^. A neighbour-joining tree revealed several breeds with ancient origins that could be separated from the remaining breeds with modern European origins^3,25,26^. The ancient breeds showed lower attachment behaviour toward their owners, suggesting that this attachment behaviour was acquired during the relatively recent domestication process. Interestingly, as even these ancient breeds with little human contact were able to use communicative signals, dogs may have acquired these abilities before the recent formation of dog breeds^23,28^. These results suggest that comparisons between and within dog breeds regarding gene–behaviour associations is useful for understanding the underlying genes responsible for dog domestication.

The analysis of genes related to the behaviour of various dog breeds holds great potential for understanding the domestication process. An investigation using genome sequencing data from a globally diverse collection of village dogs and wolves suggested that the initial selection during the early stages of dog domestication was for a behavioural trait influenced by genes associated with the perturbation of crucial neural crest signalling pathways^24^. A genome-wide polymorphism analysis of a global sample of dogs and wolves suggested that there had been selection pressure on the ‘adrenaline and noradrenaline biosynthesis pathway,’ well known for its involvement in the fight-or-flight response^29^. Other genome-wide analyses have also identified various genomic regions as candidates for affecting dog cognitive phenotypes, such as inhibitory control, sociability, and interspecies communication^30^. An analysis of the dog genome region which is known in humans to be linked to Williams–Beuren syndrome (WBS), a multisystem congenital disorder characterized by hyper-social behaviour, provided evidence that structural variants in genes previously implicated in the behavioural phenotype of patients with WBS and contained within the WBS locus, contribute to extreme sociability in dogs^8^. However, the genes associated with the skill of reading human gestures and attachment behaviour toward humans differ between dogs and wolves and are not well-understood.

Genomic research has identified a list of genes that underwent positive selection during domestication^25,31^. The functions of these candidate selected genes are diverse, including digestion, gene ontology, neurological processes, and sexual reproduction. Some of these selections may have influenced the endocrine system, resulting in domestic dogs acquiring their unique behaviours during the domestication process^15^. We focused on cortisol and OT as important hormones related to dog domestication. We hypothesized that cortisol regulation of social tolerance and non-fearful response to humans may have been the most important turning point in the domestication of dogs^15^. A decrease of cortisol can facilitate social cognitive skills in dogs, probably owing to a reduction of their fear response to humans, as suggested previously by other researchers^32^. However, cortisol suppression does not fully explain the substantial ability of dogs to understand human communicative signals and form social bonds with humans. Therefore, we focused on the OT system. We hypothesized that OT is another hormone which played a key role in the dog domestication process^15^. Another gene which is of interest is WBSCR17. WBSCR17 is in the critical region of patients with Williams–Beuren Syndrome and is predominantly expressed in the brain and heart. WBSCR17 is thought to be involved in brain development, through the O-glycosylation of neuron proteins^33^.

In this study, to explore the genes related to behavioural and cognitive changes in the dog domestication process, we investigated the association between the human-related cognitive ability of dogs and polymorphisms in the following four genes: melanocortin 2 receptor (MC2R), WBSCR17, OT, and OTR. We selected two distinctive social cognitive skills in dogs that are clearly different from wolves: the ability to follow pointing and gestures from humans, tested using a two-way choice test^13^, and gaze behaviour, tested using a problem-solving test^12^.

## Results

### Two-way choice test and problem-solving test

**Figure 1:**
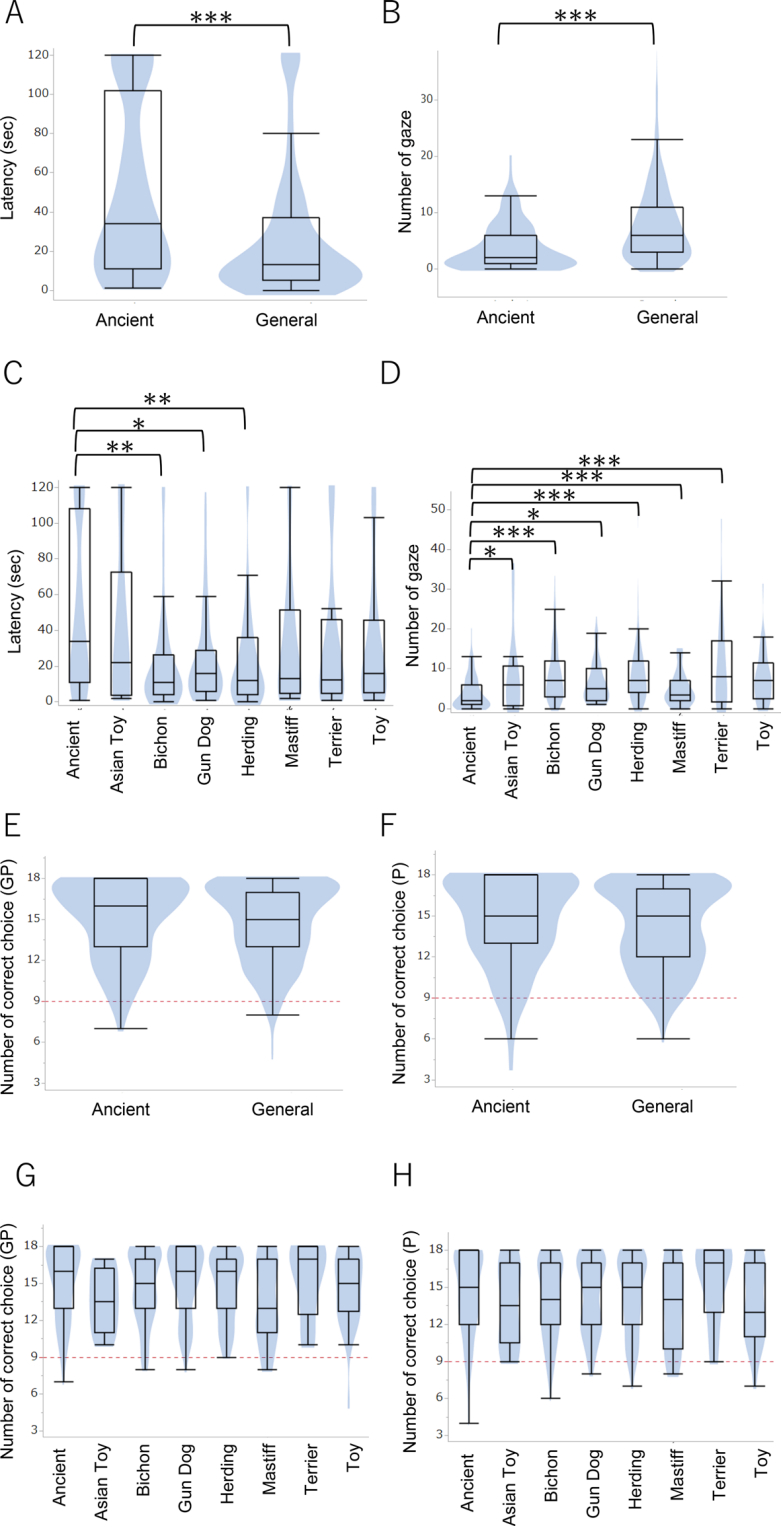
Breed comparisons of the problem-solving test (**A**–**D**) and two-way choice test (**E**–**H**). The Ancient breeds showed longer latency before looking back at the experimenters than the General breeds (**A**), particularly the Bichon, Gun Dog and Herding groups. The Ancient breeds also gazed toward the experimenters less often than the general breeds (**B**, **D**). In contrast, there was no breed difference found in the two-way choice test (**E**–**H**). *p < 0.05, **p < 0.01, ***p < 0.001 (GLM followed by Bonferroni post-hoc comparison).

We evaluated the human-related cognition ability of 624 domestic dogs (Table S1) using a two-way choice test and a problem-solving test commonly used to assess human-related cognition ability in canines. After separating the dog breeds into two groups, the Ancient group and the General group, based on their genetic distance from wolves [according to the Parker cladogram^26^], we found that the dogs in the Ancient group looked at humans less often than the dogs in the General group during the problem-solving test (Fig. 1A,B). We further separated the General group into seven subgroups (again based on their genetic distance from wolves according to the Parker cladogram) and compared all eight groups. We found that the dogs in the Ancient group looked at humans less often than those in the other groups, except for the Toy group (Fig. 1C,D). In contrast, the scores in the two-way choice test showed no significant breed-related differences (Fig. 1E–H). The effects of training, age, sex, and neutered status are summarized in Tables S5 and S6.

### Polymorphism search for the candidate genes associated with human-related cognition ability

**Figure 2:**
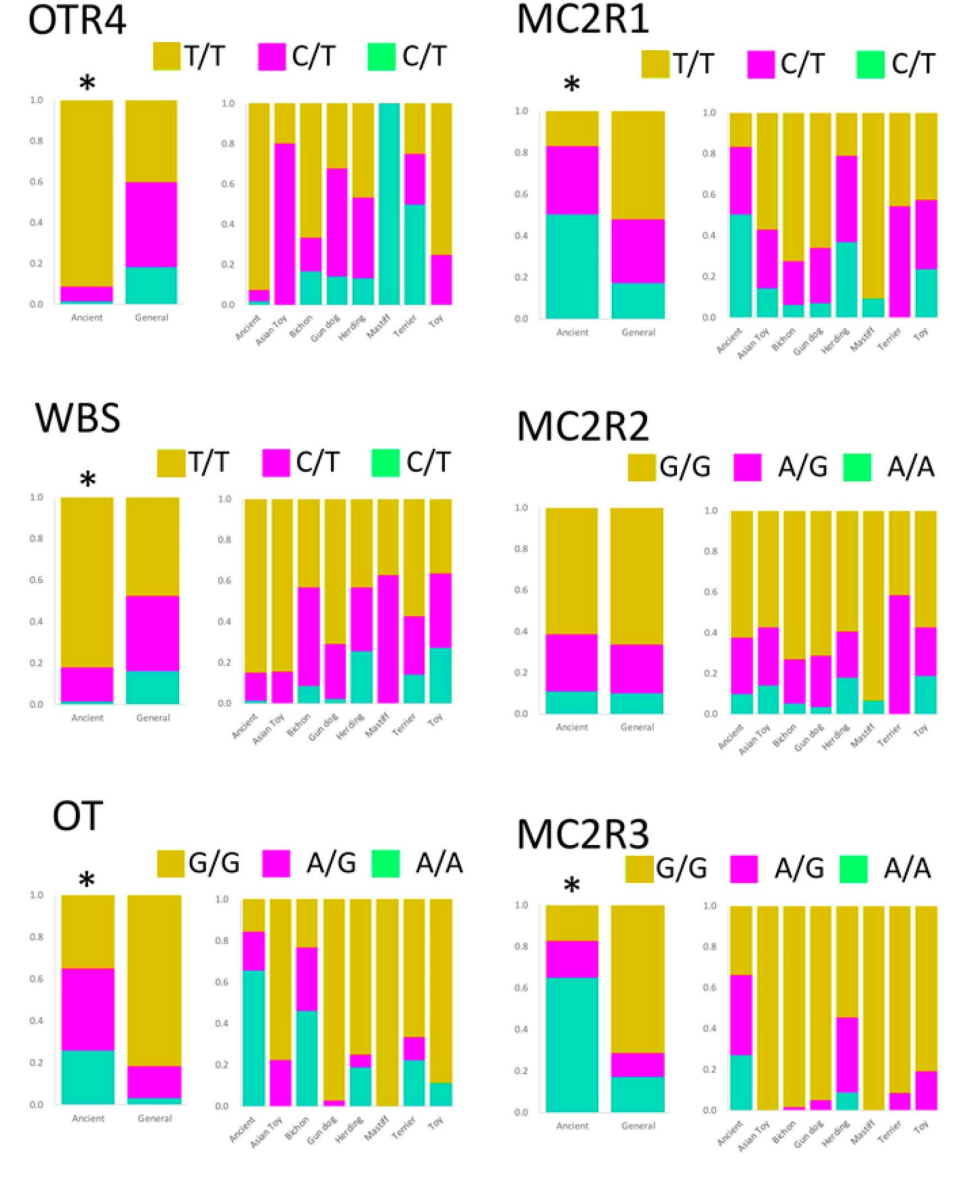
Breed comparisons of the allelic frequencies of six single-nucleotide polymorphisms (SNPs) of the candidate genes. All SNPs except MC2R SNP2 showed differences between the Ancient and General breed groups. *p < 0.05 (Chi-square test).

We identified some polymorphisms in the candidate genes, WBSCR17, MC2R, OT, and OTR, using the Ensembl and the National Centre for Biotechnology Information databases and amplified the fragments including the selected polymorphisms using PCR. We analysed the polymorphisms of the dogs using a Premix Sequence Analysis service and searched for polymorphisms with a more than a 5% difference in allele frequencies. One synonymous single-nucleotide polymorphism (SNP) of WBSCR17 (rs24319159), two synonymous SNPs (rs21898857, SNP1 and rs21898855, SNP2) and one non-synonymous SNP (c.871G > A, Val291Ile, SNP3) of MC2R, one SNP in the intron region of OT (c.322 + 50C > A), and one synonymous SNP of OTR (rs8679682) were found. We compared their frequency differences among the Ancient group and the General group, and five of these SNPs showed significant differences (Fig. 2).

### Correlations between gene polymorphisms and the scores of behaviour tests

**Figure 3:**
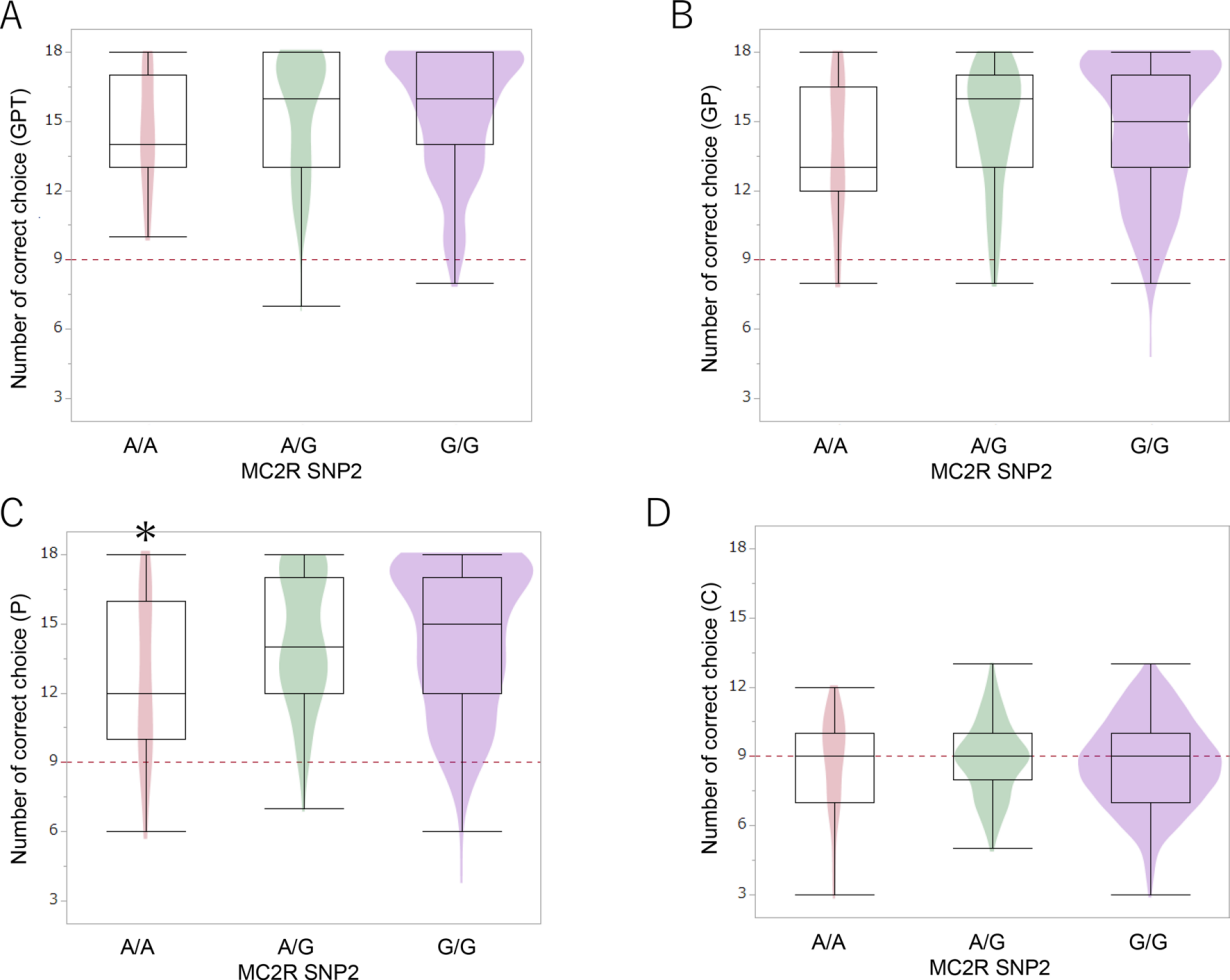
Allelic comparisons of MC2R SNP2 polymorphisms in the two-way choice test. In the pointing test (C), dogs with the A/A allele showed fewer correct choices than dogs with the A/G or G/G alleles. *p < 0.05 (GLM followed by Bonferroni post-hoc comparison).

**Figure 4:**
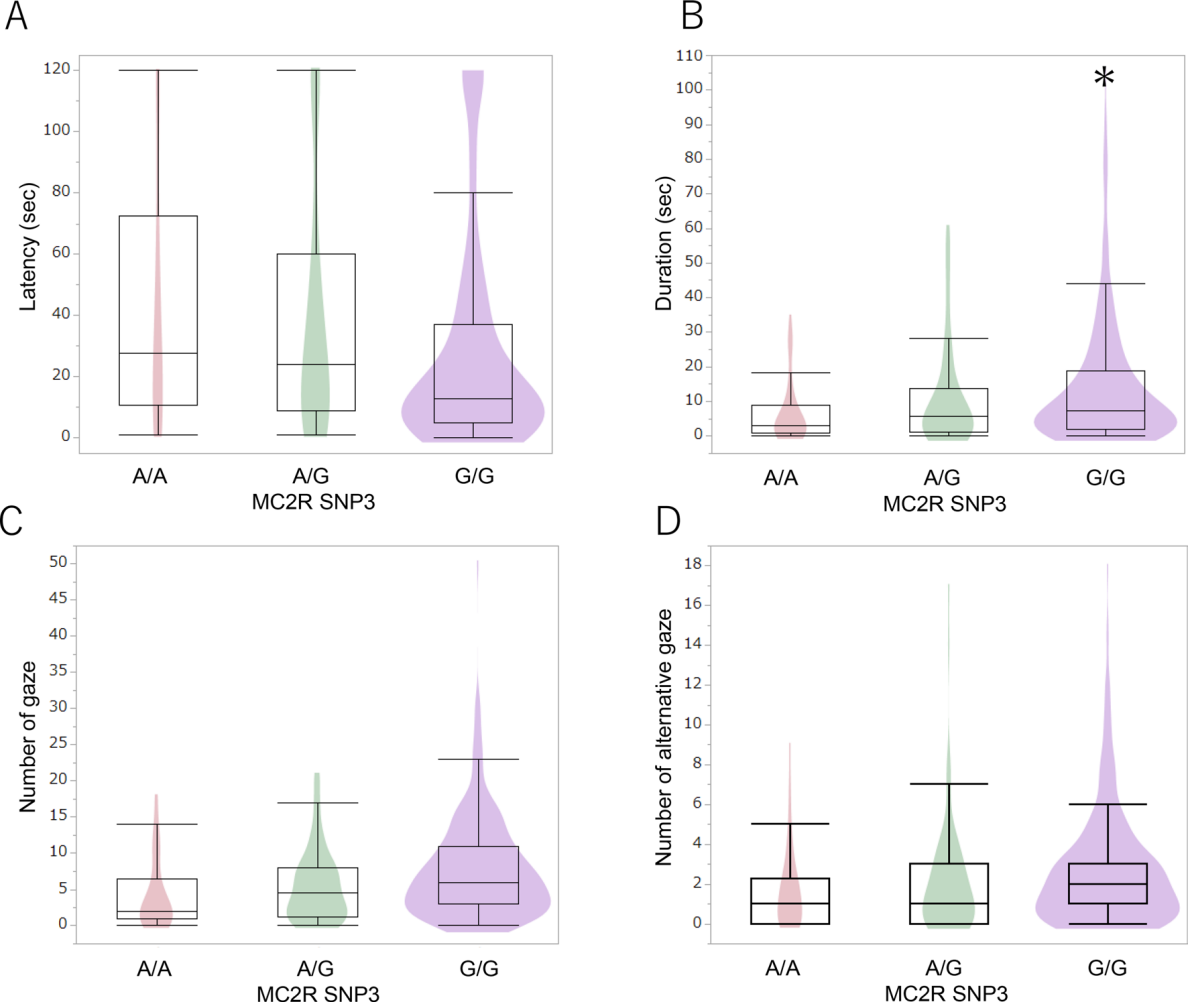
Allelic comparisons of MC2R SNP3 polymorphisms in the problem-solving test. Dogs with the G/G allele looked back at the experimenters for longer durations than dogs with the A/G and A/A alleles (B). *p < 0.05 (GLM followed by Bonferroni post-hoc comparison).

Because the Ancient and General groups showed differences in the behavioural tests, and there were significant differences in gene polymorphisms between the Ancient and General groups, we carried out generalized liner model (GLM) analysis on the scores of the behaviour tests using breed differences and polymorphisms as independent variables. The GLMs revealed one SNP associated with the scores of the two-way choice test, and six SNPs associated with the scores of the problem-solving test (Table S3). The OTR SNP associated with the duration and number of gaze events and gaze-alternation in the problem-solving test. The WBSCR17 SNP associated with the number of gaze events in the problem-solving test. MC2R SNP1 associated with the duration and number of gaze events in problem-solving test. MC2R SNP2 associated with the correct choice rate in the pointing test (Fig. 3, Tables S3, S4) and gaze-alternation in the problem-solving test. MC2R SNP3 associated with the duration and number of gaze events and gaze-alternation in problem-solving test (Fig. 4, Tables S3, S4). Finally, the OT SNP associated with latency and gaze-alternation in the problem-solving test (Tables S3, S4). We analysed whether training experience, age and sex could influence these correlations. Training experience and sex found no bias in any of the SNPs. As for OTR SNP, dogs of C/T genotype showed higher age as compared to the other two genotypes. As for MC2R SNP1, dogs of C/G genotype showed higher age as compared to the C/C genotypes (Table S6). There was also no correlation between the results of the two-way choice test and the problem-solving test (Fig. S1).

## Discussion

As mentioned above, we carried out a two-way choice test and a problem-solving test to study human-related cognition ability in dogs. Although differences were identified between the Ancient and General groups in the problem-solving test, there were no discernible differences in the two-way choice test. The Ancient group we studied was composed almost entirely of Japanese breeds and therefore, we are unable to say whether this would be true for Ancient breeds from other countries. In addition, there could be the influence of recent selection on the breed-typical behavior. However, the problem-solving test in our study, combined with the result of earlier genome-wide studies of the domestic dogs^7,25,26^, indicates that Japanese dogs which have relatively close DNAs to wolves show behaviour similar to wolves. The results of the two-way choice test were similar in both groups of dogs, showing that both groups have similar capabilities in understanding human gestures and adjusting their responses accordingly. In contrast, the General group dogs showed higher eye-contact behaviour in the problem-solving test. This suggests that the capability of dogs to understand human commands and adjust their own behaviour accordingly, as tested by the two-way choice test, evolved early in the domestication of dogs. The stronger tendency of gazing at humans, as tested by the problem-solving test, was a desirable characteristic that was intentionally chosen for, through human-selected breeding, after dogs had evolved.

In a different study by our group, we achieved results indicating that dogs in the General group had a stronger attachment to their owners than dogs in the Ancient group; however, they showed similar degrees of fear and aggression^34^. The current hypothesis is that ancient wolf individuals that had low levels of fear and aggression started to approach human areas and evolved into dogs^32^. Earlier studies tried to recreate this process using foxes^16^. As wolves and foxes are different species, the results of the fox study cannot be used as a direct comparison for the domestication of dogs; however, it is an interesting case study using a similar canid species. Within a short period (approximately 40 years), the foxes that were chosen and bred for low aggression toward humans showed higher positive scores in a two-way choice test^35^. This suggests that the ability to understand human commands may have evolved early in the domestication of dogs. Our results showed that MC2R SNP2 was correlated with the correct choice rate in the two-way choice test; therefore, an association between the hormone which is widely known as positively related to anxiety and social avoidance, and the ability of dogs to adjust their own behaviour based on human commands under the two-way choice test was supported.

However, the dogs in the Ancient group scored lower in their tendency to look at human faces for instruction, as tested by the problem-solving test, and in their level of attachment, than dogs in the General group. Although both the two-way choice test and the problem-solving test require the dogs to look at humans, in the two-way choice test the human commands were automatically provided, whereas the problem-solving test, it required the dogs to seek out the connection with humans. It may have become necessary through the process of domestication for dogs to gaze at humans for instruction and initiate communication to build a more successful relationship between humans and dogs. Furthermore, as even stray dogs that were not brought up in human households show this characteristic, it has been previously suggested that there is a genetic component involved^36,37^ and our results support this hypothesis.

To make the complicated process of dog domestication clearer, various aspects such as genetics, hormones, and behaviour should be studied, and the correlations between the results of the two-way choice test and the problem-solving test and genetic polymorphism are an important step in understanding the biological basis of dog–human social behaviour. We found several genetic polymorphisms that seem to correlate with the results of the two-way choice test and problem-solving test. Of these, we have focused on the MC2R genetic polymorphisms (MC2R SNP2 and MC2R SNP3). MC2R codes the adrenocorticotropic hormone receptor in the adrenal glands and is involved in the production of cortisol. In the fox domestication study we referred to earlier, the activity levels of the hypothalamic–pituitary–adrenal axis and the blood cortisol levels decreased as the foxes were selectively bred for lower levels of fear and aggression^16^. MC2R SNP2 correlated with the two-way choice test and MC2R SNP3 correlated with the problem-solving test. MC2R SNP2 and MC2R SNP3 are located close together on the 3′ end of the strand. MC2R SNP2 is a synonymous mutation of alanine, whereas the newly identified MC2R SNP3 is a non-synonymous mutation of valine and isoleucine. The MC2R protein, encoded by the MC2R gene, forms seven transmembrane receptors. The C-terminus, where MC2R SNP2 and MC2R SNP3 polymorphic sites are located, is important for the migration of these receptors to the cell surface^38^. MC2R SNP2 is a synonymous mutation, but whether it affects gene expression or only displays apparent relevance owing to its association with another gene is unclear. As for MC2R SNP3, it is possible that it directly affects melanocortin receptor functioning as a non-synonymous mutation; however, the details are also unclear. We are considering comparing these polymorphisms with the amount of cortisol in our future research. Finally, the two-way choice test and the problem-solving test showed correlations with polymorphisms of the same gene; however, the two tests were not correlated with each other. This implies that the biological basis of the two-way choice test and the problem-solving test are genetically and/or evolutionarily independent.

The link between OT or OTR and the social cognitive abilities of dogs have been reported in several studies. Kis et al. showed that OTR polymorphisms were correlated with proximity seeking and friendliness behaviour^19^. Similar results have been found for greeting behaviours toward humans^39^. OTR polymorphisms are also known to be correlated with the effectiveness of OT administration in the human-seeking behaviour task in dogs^40^. As these tests studied factors such as affinity and proximity seeking, which were not used in our study, the results cannot be directly compared; however, it is interesting that, in our study, the OT and OTR polymorphisms were found to be associated with the results of the problem-solving test. Dogs were found to direct their gaze toward humans, an important social signal for humans, and humans were found to secrete OT in response, leading to attachment behaviour^10^. It is possible that looking behavior has an important function in initializing and maintaining communicative interaction, thereby explaining the correlation with OT and OTR genes. The WBSCR17 gene does not directly affect the endocrine system, but it is known to be associated with domesticated behaviour in dogs^8^. However, the behaviour associated with WBSCR17 was hyper-sociability with humans and was therefore different from what we tested in the current study. As it seems likely that WBCSR17 was also associated with the early stages of domestication, such as ancient wolves initially approaching humans, it is necessary to clarify the role of these genes by diversifying behavioural experiments. Two genetic polymorphisms had age biases that can influence the behavioural parameters in the Problem-solving test. Due to being not enough degree of freedom, the comprehensive statistical analysis was not possible, and this is an issue for future work.

The polymorphism of the endocrine genes that we focused on and the clearly distinct social cognitive skills of dogs and wolves indicate that a change in endocrine functioning was associated with the advancement of these cognitive skills^15^. To elucidate the correlations between the identified gene types and the behavioural phenotypes, it will be necessary to study the developmental processes of dogs and wolves and measure their endocrinal dynamics after development^41^. Furthermore, such complex social cognitive skills cannot be completely explained by just the identified genes and must be controlled by multiple genes; therefore, it is necessary to search for other genes and verify their overall effects. In sum, we tested 624 dogs and searched the candidate genes responsible for communication with humans, the MC2R gene was the most effective to the skill of dogs in two-way choice test and problem-solving task, indicating that this gene can be mutated in the early domestication process of dogs.

## Materials and methods

### Experimental design

We tested 624 dogs socialized to humans as house dogs (Table S1). All were recruited voluntarily through their owners. The dogs were individually tested, either in an indoor location or an outdoor location with quiet surroundings. The procedures of the experiments were approved by the Animal Ethical Committee of Azabu University (#130,304–2). We confirmed that all methods were carried out in accordance with the guideline of Ministry of Education, Culture, Sports, Science and Technology, Japan, and all methods were reported in accordance with ARRIVE guidelines. Before starting the experiments, informed consent was obtained from the owners for inclusion of their dogs in the study.

### Behavioural data

#### Two-way choice test

The test apparatus and the procedure were similar to those previously reported^13^. The test apparatus consisted of polystyrene foam blocks with a wooden board (90 cm long) placed on top. Food was hidden underneath one of two bowls (10 cm diameter) placed at opposite ends of the wooden board.

The test consisted of two phases, the warm-up and the experimental session. In the warm-up, the subject was positioned in front of the apparatus. Food was hidden underneath one of the bowls. The experimenter said "ok," allowing the subjects to choose one of the bowls by touching it. If the subjects touched the baited bowl first, they were allowed to eat the hidden food treat. If the subjects touched the empty bowl first, they were shown that it was empty. The experimental session began after the subject could reliably find the hidden food (successfully chose the baited bowl three times in a row). If the subject did not pass the warm-up session in 10 min (could not choose the baited bowl three times in a row in 10 min), they were omitted from the rest of the test.

In the experimental session, the experimenter indicated the location of the hidden food using the following cues: (1) a Gaze + Point + Tap cue (GPT)-the experimenter looked toward the baited bowl while extending their cross-lateral arm and tapping on it three times, making a small noise; (2) a Gaze + Point cue (GP)-identical to the Gaze + Point + Tap cue, except that the tapping was replaced with pointing at the baited bowl (index finger 10–15 cm from the bowl); (3) a Point cue (P)-identical to Gaze + Point cue, except that no gaze cue was given (the experimenter looked at the subject); and (4) a control cue (C)-the experimenter gave no cue (looked straight ahead). The subject was allowed to choose one of the bowls and the number of correct responses was recorded. A subject received 18 trials in each of the four conditions, for a total of 72 trials (54 experimental trials followed by 18 control trials). The behaviour was also video-recorded.

#### Problem-solving test

The test apparatus and the procedure were similar to the bin-opening test performed by Miklosi et al.^12^. The test apparatus consisted of a wooden board (24 × 45 × 2 cm) with a plastic container placed on top. In the warm-up procedure, the subjects were given the opportunity to learn how to solve the problem situation in six repeated trials. After the subjects had learned the task (opened the container which contained a piece of food), the subjects underwent the test. In the test session, the experimenter presented the subjects with the same problem, but this time the problem was unsolvable (the plastic container was closed mechanically). The behaviour of the subjects was video-recorded and analysed for 2 min. The latency, duration, number of gazing behaviour events (gaze directed toward the experimenters), and number of gaze alternations between the experimenters and the bin were recorded.

### Genetic analysis

Blood samples were collected from the dogs by veterinarians. Genomic DNA was isolated using the QIAamp® DNA Mini Kit (QIAGEN, Hilden, Germany). The numbers of dogs of each breed are shown in Table S1. Polymorphisms were searched using the Ensembl and National Centre for Biotechnology Information databases. The fragments including the selected polymorphisms were amplified by PCR using the primer pairs shown in Table S2. The PCR reactions were performed with KOD-FX Neo DNA polymerase (TOYOBO, Osaka, Japan) in a PCR Thermal Cycler Dice Touch (Takara, Shiga, Japan). The samples were initially denatured at 94 °C for 2 min, followed by 40 cycles of 98 °C for 10 s and 65 °C for 30 s, and a final extension at 72 °C for 7 min. The PCR products were purified using Nucleo Spin® Gel and a PCR Clean-up Kit (Takara) after being confirmed using 1.5% agarose gel electrophoresis. The samples were shipped to the Takara CDM Centre (Mie, Japan) and subjected to Premix Sequence Analysis (Takara).

### Statistical analysis

The dogs were separated into eight breed groups: (1) Ancient, (2) Asian Toy, (3) Companion and Toy, (4) Bichon and Poodle, (5) Terrier, (6) Scent Hound and Gun Dog, (7) Sight Hound and Herding, and 8) Mastiff, following Parker’s (2017) cladogram (Table S1). Other dog breeds not shown in Parker’s cladogram were eliminated from further analyses. The genetic polymorphism data were compared between the Ancient, General, and other breed groups using chi-square tests.

The distribution of some of the behavioural data did not follow a normal distribution. GLMs were performed using a Poisson distribution for the two-way choice test, and for the numbers of gazing events and gaze alternations in the problem-solving test. An exponential distribution was used for the analysis of latency and duration in the problem-solving test (JMP® ver. 14, SAS Institute Inc., Cary, NC, USA).

## Supplementary Information

Supplementary Information.

## Data Availability

All other data are available from the corresponding author on request.
