# Supplementary material for: Identification of genes associated with human-canine communication in canine evolution
Source: Sci Rep. 2022 Jun 9;12:6950. doi: 10.1038/s41598-022-11130-x (PMC9184530; doi:10.1038/s41598-022-11130-x)
Supplement: Supplementary file 1 — Supplementary Information. [file 41598_2022_11130_MOESM1_ESM.docx]

Supplementary Materials for

Identification of genes associated with human-canine communication in canine evolution.

Akiko Tonoike, Ken-ichi Otaki, Go Terauchi, Misato Ogawa, Maki Katayama, Hikari Sakata, Fumina Miyasako, Kazutaka Mogi, Takefumi Kikusui, and Miho Nagasawa*

*Corresponding author. Email: nagasawa@azabu-u.ac.jp

**This PDF file includes:**

Fig. S1

Table S1–S6


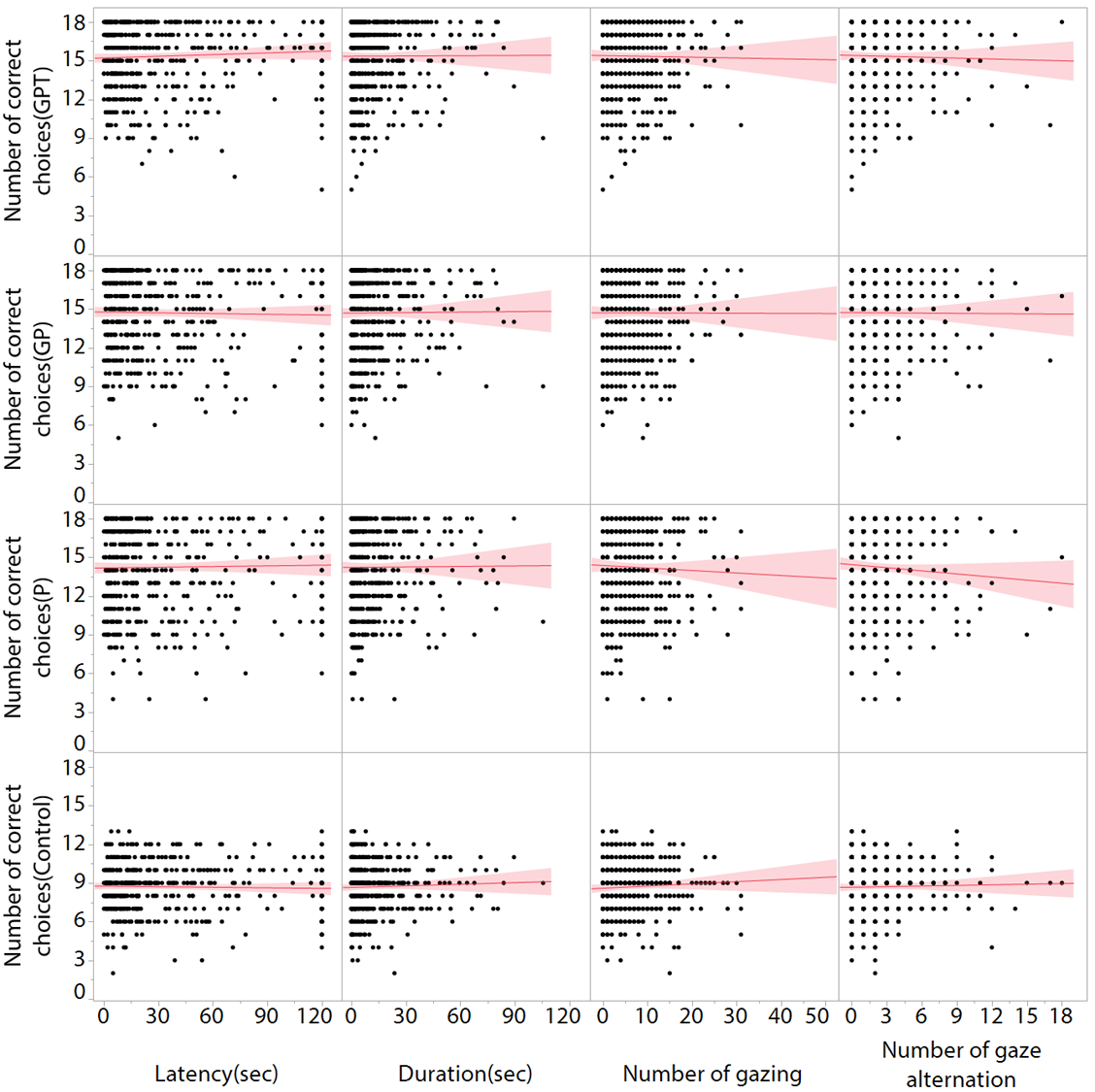


**Fig. S1.**

Correlation analysis of the behavioral parameters in the two tests (problem solving test and two-way choice test). There were no correlations between them.**Table S1.**

**The number of dogs in each breeds and the genetically clustered breed groups used for the cognition tests.**

**Table S2.**

**Primers used for PCR amplification to genotype the dog gene polymorphisms.**

**Table S3.**

**Summary of GLM analysis of Breed groups and Genetic polymorphisms**

(continued to next page)

**Table S4.**

**Post-hoc tests of GLM analysis of Breed groups and Genetic polymorphisms**

(continued to next page)

**Table S5.**

**Summary of GLM analysis of basic information of the dogs**

**Table S6.**

**Summary of Chi-squared test of Genetic polymorphisms and Training experience**
